# Sulforaphane inhibits growth of phenotypically different breast cancer cells

**DOI:** 10.1007/s00394-013-0499-5

**Published:** 2013-02-07

**Authors:** Anna Pawlik, Aleksandra Wiczk, Angelika Kaczyńska, Jędrzej Antosiewicz, Anna Herman-Antosiewicz

**Affiliations:** 1 Department of Molecular Biology, University of Gdańsk, Wita Stwosza 59, 80-308 Gdańsk, Poland; 2 Department of Bioenergetics and Physiology of Exercise, Medical University of Gdańsk, Dębinki 1, 80-211 Gdańsk, Poland

**Keywords:** Breast cancer, Sulforaphane, PI3K-AktmTOR-S6K1 pathway, Autophagy

## Abstract

**Purpose:**

Cancer development and resistance to chemotherapy correlates with aberrant activity of mitogenic pathways. In breast cancers, pro-survival PI3K-AktmTOR-S6K1 signaling pathway is often hyperactive due to overexpression of genes coding for growth factors or estrogen receptors, constitutive activation of PI3K or Akt and loss of PTEN, a negative regulator of the pathway. Since epidemiologic as well as rodent tumor studies indicate that sulforaphane (SFN), a constituent of many edible cruciferous vegetables, might be a potent inhibitor of mammary carcinogenesis, we analyzed the response of four breast cancer cell lines representing different abnormalities in ErbB2/ER-PI3K-AktmTOR-S6K1 signaling pathway to this compound.

**Methods:**

Four different breast cancer cell lines were used: MDA MB 231, MCF-7, SKBR-3 and MDA MB 468. Cell viability and ultrastructure, protein synthesis, autophagy induction and phosphorylation status of Akt and S6K1 kinases upon SFN treatment were determined.

**Results:**

We observed that all four cell lines are similarly sensitive to SFN. SFN decreased phosphorylation of Akt and S6K1 kinases and at higher concentrations induced autophagy in all studied cell lines. Moreover, global protein synthesis was inhibited by SFN in investigated cell lines in a dose-dependent manner.

**Conclusion:**

These results indicate that SFN is a potent inhibitor of the viability of breast cancer cells representing different activity of the ErbB2/ER-PI3K-AktmTOR-S6K1 pro-survival pathway and suggest that it targets downstream elements of the pathway.

## Introduction

Human cancers frequently display abnormalities in signaling pathways that regulate cell proliferation and survival. In breast cancer, there is an increasing recognition of the pivotal role played by the growth factor receptors-PI3K-Akt-mTOR-S6K1 pathway. The ErbB2 receptor is overproduced in approximately one-third of breast tumors, and it correlates with poor clinical prognosis [1, 2]. Breast cancer therapies targeted against ErbB2, although very specific, do not apply to all patients as some cells become resistant. It often correlates with hyperactivation of PI3K, Akt and S6K1 kinases or mutations in suppressor gene coding for PTEN phosphatase [3]. Reduced PTEN protein expression was seen in 38 % of invasive cancers and in 11 % of in situ breast cancers [4]. Moreover, estrogen receptor (ERα) activation can directly drive the PI3K-Akt pathway. Constitutive activation of Akt is associated with the resistance to either tamoxifen treatment or estrogen deprivation in hormone-dependent cancers [5]. Thus, targeting the critical downstream members of the pathways that cells remained dependent upon might be the way to overcome their resistance. Indeed, preclinical studies have shown that the mTOR antagonists can restore endocrine sensitivity in breast cancer cells [6].

Cruciferous plants are a rich source of bioactive isothiocyanates, including sulforaphane (SFN). It has been demonstrated that SFN or its precursor, glucoraphanin, reduced the number, size and rate of development of mammary tumors in rats treated with dimethylbenz[*a*]anthracene [7, 8]. SFN decreased the DNA-adduct formation in normal mammary cells exposed to polycyclic aromatic hydrocarbons [9]. Chemopreventive activity of SFN is related to induction of enzymes responsible for mutagens elimination [7, 8, 10] and inhibition of phase I enzymes, which activate pro-carcinogens [11]. SFN also induces cell cycle arrest and apoptosis of neoplastic cells [12–17]. Among reported mechanisms underlying anticancer activity of SFN, there are oxidative stress induction [14, 18, 19], DNA damage checkpoint activation [13], inhibition of histone deacetylases [16, 20] or direct binding to cellular proteins, such as tubulins [21]. It has been shown that SFN-induced block in mitosis of human MCF-7 and murine F3II mammary cancer cells correlated with cdk1 activation, cyclin B1 accumulation and disruption of mitotic microtubules polymerization [15, 22]. G2/M cell cycle arrest as well as apoptosis induction have been observed in a panel of different breast cancer cell lines treated with SFN [16]. Interestingly, pathways leading to apoptosis were cell line specific with induction of Fas ligand and caspase 8 in MDA MB 231 cells and activation of mitochondrial pathway in MDA MB 468, T47D and MCF-7 cell lines [16].

In our study, we addressed the question whether SFN affects pro-survival PI3K-Akt-mTOR-S6K1 signaling pathway. Thus, we tested sensitivity to SFN of four established breast cancer cell lines which have distinct characteristics regarding the status of the pathway elements: MDA MB 231 is estrogen-independent cell line with low level of ErbB2 and EGFR1 receptors, MCF-7 is estrogen receptor (ER) positive cell line with low level of ErbB2 and EGFR1 receptors, MDA MB 468 cells are devoid of PTEN repressor (negative regulator of the PI3K-Akt-mTOR signaling) and possess high EGFR1 level and SKBR-3 cells overproduce receptor kinase ErbB2 [23–25] (Table 1). Our results indicate that, despite distinct signaling at the level of receptors, SFN decreases survival of all tested cell lines in a similar rate. Drop in phosphorylation of Akt and S6K1 kinases, inhibition of protein synthesis and autophagy induction strongly indicate that SFN targets elements of the pathway being downstream of receptors.

**Table 1:** Phenotypic characteristics and sensitivity to SFN (IC50) of breast cancer cell lines

Cell line	Presence of ERα	Overexpression of ErbB2	Overexpression of EGFR1	Presence of PTEN	IC50 (μM)
MDA MB 231	−	−	−	+	21
MCF-7	+	−	−	+	19
MDA MB 468	−	−	+	−	20
SKBR-3	−	+	−	+	25

## Materials and methods

### Reagents

D,L-SFN (CH_3_-SO-(CH _2_)_4_-NCS, purity 99 %) was purchased from LKT Laboratories (St. Paul, MN). Tissue culture media, penicillin/streptomycin antibiotic mixture and fetal bovine serum were from GIBCO (Grand Island, NY). DMSO, sodium pyruvate, thiazolyl blue tetrazolium bromide (MTT), the anti-β-actin, anti-mouse and anti-rabbit antibodies conjugated with HRP were from Sigma (St. Louis, MO). The antibodies against p-Akt (Ser-473) and p-S6K1 (Thr-389) were from Cell Signaling Technology (Danvers, MA).

### Cell lines and cell culture

Monolayer cultures of MCF-7 cells were maintained in RPMI 1640 medium supplemented with 10 % (v/v) fetal bovine serum and antibiotics. SKBR-3 and MDA MB 468 cells were cultured in MEM containing 10 or 5 % FBS, respectively, and antibiotics. MDA MB 231 cells were maintained in MEM supplemented with 10 % FBS, 1 mM sodium pyruvate, non-essential amino acids and antibiotics. Each cell line was maintained at 37 °C in a humidified atmosphere with 5 % CO_2_. D,L-sulforaphane was prepared in DMSO (solubility 20 mg/ml) and stored at a stock concentration of 10 mM at −20 °C.

### Cell viability assay

Cell viability was determined by MTT method. Cells were seeded at a density of 4 × 10^3^ per well of 96-well plate and allowed to attach overnight. The medium was replaced with fresh medium supplemented with DMSO (0.2 % v/v), 5, 10, 20 or 30 μM SFN for 24 h. Before the end of treatment, 25 μl of MTT solution (4 mg/ml) was added to each well. After 3 h of incubation, medium was removed and formazan crystals were dissolved in 100 μl of DMSO. Absorbance was measured at 570 nm (with reference wavelength 620 nm) in Victor^3^ microplate reader. Data were obtained from at least three independent experiments, each treatment condition assayed in triplicate.

### Detection of LC-3 localization

Cells (3.5 × 10^5^/well of 6-well plate) were grown on coverslips and allowed to attach overnight. The cells were transfected with pGFP-LC3 plasmid DNA, kindly provided by Dr. Tamotsu Yoshimori (National Institute of Genetics, Yata 1111 Mishima, Shizuoka-ken, Japan), using FuGene 6 (Roche Diagnostics) according to manufacturer recommendations. After 24 h, cells were exposed to 40 μM SFN or equal volume of Me_2_SO (final concentration 0.4 % v/v) for 6 h at 37 °C. After this time, cells were washed with PBS and fixed at 37 °C for 0.5 h using 2 % paraformaldehyde. Slides were mounted and analyzed under a Nicon Eclipse E800 fluorescence microscope.

### Transmission electron microscopy

To determine the effect of SFN treatment on autophagy induction, transmission electron microscopy was performed, as described previously [26] with some modifications. Briefly, cells (1.5 × 10^5^) were plated in 12-well plates and allowed to attach overnight. The cells were then treated with either 40 μM SFN or equal volume of vehicle, Me_2_SO (final concentration 0.4 % v/v) for 6 h at 37 °C. The cells were fixed in ice-cold 2.5 % electron microscopy grade glutaraldehyde in 0.1 M PBS (pH 7.4). The specimens were rinsed with PBS, post-fixed in 1 % osmium tetroxide with 1 % potassium ferricyanide, dehydrated through a graded series of ethanol (30–100 %) and embedded in Epon, Fluka (USA). Semi-thin (300 nm) sections were cut using a RMC Power Tome XL ultramicrotome, stained with 0.5 % toluidine blue and examined under a light microscope. Ultrathin sections (65 nm) were stained with 2 % uranyl acetate and Reynold’s lead citrate and examined on a Philips CM100 transmission electron microscope.

### Immunoblotting

Cells were treated with SFN as described above and lysed using a solution containing 50 mM Tris, 1 % Triton X-100, 150 mM NaCl, 0.5 mM EDTA, protease and phosphatase inhibitor cocktails (Roche Diagnostics). The lysates were cleared by centrifugation at 14,000 rpm for 15 min. Lysate proteins were resolved by SDS–polyacrylamide gel electrophoresis (SDS-PAGE) and transferred onto PVDF membrane. The membrane was blocked with a solution containing 10 mM Tris (pH 7.4), 150 mM NaCl, 0.1 % Tween-20 and 5 % non-fat dry milk and incubated with the desired primary antibody over night at 4 °C. The membrane was treated with appropriate secondary antibody for 1 h at room temperature. The immunoreactive bands were visualized by enhanced chemiluminescence reagent (Thermo Scientific Pierce, Rockford, IL). The blots were stripped and reprobed with anti-β-actin antibody to normalize for differences in protein loading. The change in protein level was determined by densitometric analysis of the immunoreactive bands by Quantity One software (BioRad) followed by correction for the respective loading control. The immunoblotting for each protein was performed at least twice using independently prepared lysates.

### Protein synthesis assay

Cells (1.5 × 10^5^/well) were cultured in 12-well plates and treated with 10, 20, 40 μM SFN for 3 h and 2 μCi/well L-[3, 4, 5-^3^H]-leucine. Both, floating and attached cells were collected, fixed in 5 % TCA at room temperature for 30 min and washed with 5 % TCA for 15 min. The acid-insoluble material was dissolved in 0.1 mol/L KOH overnight at 4 °C, and aliquots were used to determine the radioactivity using liquid scintillation counter (Beckman LS3133P). Radioactivity of samples was normalized to cell number.

### Statistical analysis

Data were analyzed using GraphPad Prism software. One-way ANOVA followed by Dunnett’s multiple comparison test was used to determine statistical significance of difference in measured variables between control and treated groups. Difference was considered significant at *P* < 0.05.

## Results

### Sulforaphane is a potent growth inhibitor of phenotypically different breast cancer cell lines

**Fig. 1:**
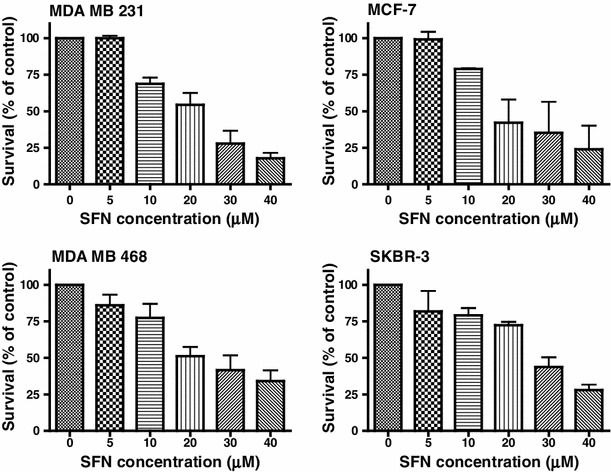
Sulforaphane decreases viability of phenotypically different cells in a dose-dependent manner. MDA MB 231, MCF-7, MDA MB 468 and SKBR-3 cells were treated with DMSO (0) or different concentrations of SFN (5, 10, 20, 30 or 40 μM) for 24 h. Their viability was assayed by MTT method as described in “Materials and methods”. Each point is mean (±SE) of three experiments done in triplicate

We examined the effects of SFN on viability of four breast cancer cell lines: MDA MB 231, MCF-7, MDA MB 468 and SKBR-3. These cell lines have been chosen as they represent examples of different abnormalities in PI3K-Akt-mTOR-S6K1 signaling pathway (Table 1). MTT assay revealed that SFN inhibited viability of all cell lines in a dose-dependent manner (Fig. 1). Moreover, anti-proliferative activity of SFN was similar in the case of all four cell lines, regardless of their PI3K-Akt-mTOR-S6K1 status, with IC_50_ ranging from 19 μM (for MCF-7) to 25 μM (for SKBR-3) after 24 h of treatment (Table 1).

### Sulforaphane induces autophagy in breast cancer cell lines

**Fig. 2:**
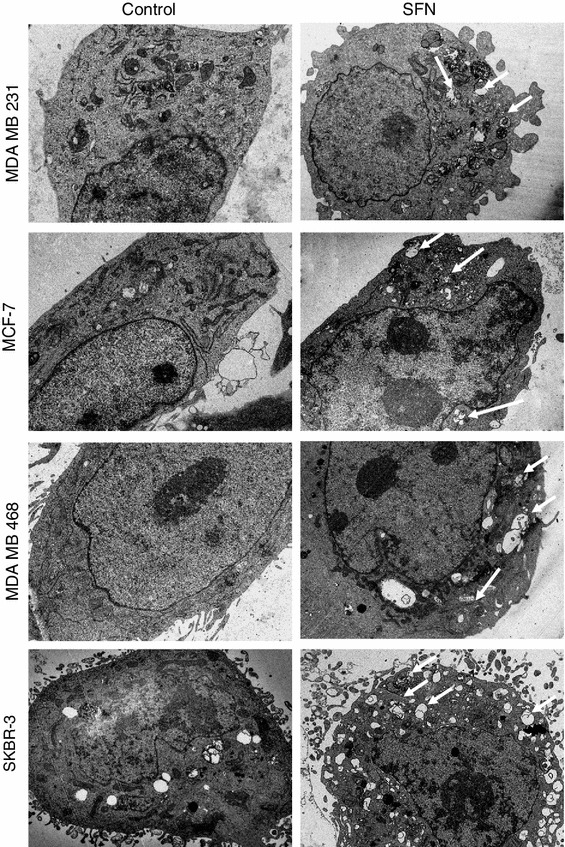
Ultrastructure of breast cancer cells in transmission electron microscopy treated or not with 40 μM SFN for 6 h. *Arrows* indicate autophagosomal vacuoles. Magnification ×1,650

**Fig. 3:**
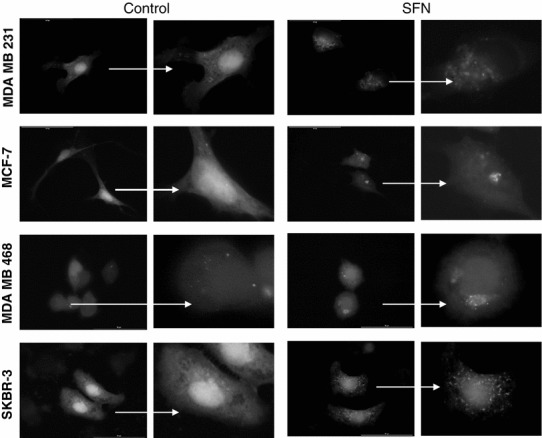
Autophagy induction by SFN in breast cancer cells revealed as punctuate localization of GFP-LC3, marker of autophagy. Cells were treated with DMSO (control) or 40 μM SFN for 6 h. Magnification ×1,000 (*left panel* in control and SFN group); on the *right panel,* enlarged respective cell is shown

It has been previously shown that SFN induces autophagy in prostate cancer cells, which precedes and delays apoptotic cell death [27]. Autophagy is an evolutionary conserved, lysosome-mediated process for degradation and turnover of long-lived proteins and whole organelles. The process begins with the formation of double-membrane vacuoles (autophagosomes) that engulf cytoplasmic material. Autophagosomes fuse with lysosomes where the inner membrane as well as the content is degraded. We sought to determine whether SFN induces autophagy in phenotypically different breast cancer cells. We examined the ultrastructures of control and SFN-treated cells by transmission electron microscopy. As can be seen in Fig. 2, SFN treatment promotes the formation of vacuoles filled with cytoplasmic material or showing different stages of its degradation in all four breast cancer cell lines. The number of membranous vacuoles is the highest in SKBR-3 cell line. To confirm that SFN-induced vacuolization is due to autophagy, we examined localization of LC3 protein in cells transiently transfected with plasmid encoding GFP-LC3 and treated or not with SFN. LC3 exists in two forms: as a cytosolic protein (LC3-I) which is evenly distributed in cytoplasm and as a processed form bound to autophagosomal membranes (LC3-II). Fluorescent microscopy of cells expressing GFP-LC3 revealed diffuse localization of the fusion protein in the case of cells treated with DMSO (Fig. 3). However, treatment with SFN induced punctuate pattern of GFP-LC3 fluorescence indicating recruitment of LC3-II to autophagosomes (Fig. 3).

### Sulforaphane decreases phosphorylation of S6K1 and Akt kinases

**Fig. 4:**
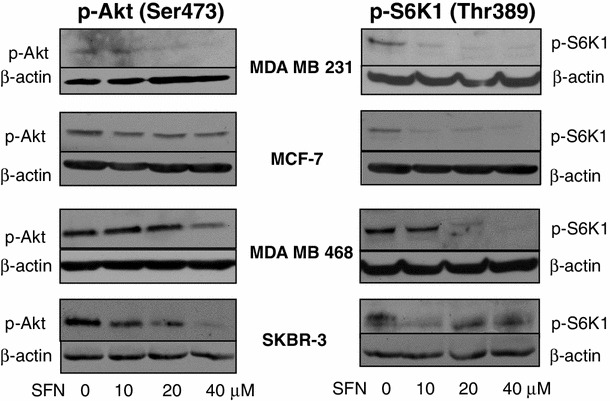
SFN decreases phosphorylation level of Akt and S6K1 kinases in all studied cell lines. Immunoblotting for p-S6K1 (Thr389) and p-Akt (Ser473) using lysates from MDA MB 231, MCF-7, MDA MB 468 and SKBR-3 cells treated with different concentrations of SFN for 3 h. The blots were stripped and reprobed with anti-β-actin antibody to ensure equal protein loading

Experimental evidence exists that mTOR is a negative regulator of autophagy [28]. Given the ability of SFN to induce autophagy in breast cancer cell lines, we hypothesized that it can negatively influence activity of mTOR. Thus, we analyzed the phosphorylation level of mTOR substrate, p70S6K1 (S6K1) at Thr389, in four breast cancer cell lines treated with different concentrations of SFN. Immunoblotting experiments revealed that SFN inhibits phosphorylation of S6K1 even at the lowest used concentration. In case of MDA MB 231 and MCF-7 cells, SFN decreased p-Thr389-S6K1 level by 75 % at 10 μM concentration and by 80 % at higher concentrations of the compound. In MDA MB 468 cells, 10 μM SFN inhibited phosphorylation of S6K1 by only 10 % but 20 and 40 μM SFN—by 80–90 %. In SKBR-3 cells, phosphorylation of S6K1 was inhibited by about 50 % in case of all studied concentrations of SFN (Fig. 4).

The mTOR is negatively regulated by TSC1/TSC2 complex. Signal about growth factors availability activates mTOR through PI3K-Akt-mTOR pathway, where Akt kinase phosphorylates TSC2 resulting in TSC1/TSC2 inactivation [29]. We sought to determine whether SFN affects phosphorylation of Akt at Ser473, position crucial for activation of this kinase. Western blotting using lysates from breast cancer cells treated with DMSO or different concentrations of SFN shows that the level of p-Ser473-Akt decreased gradually with increasing concentration of SFN in MDA MB 231 (70, 50 and 10 % of control cells in cells treated with 10, 20 and 40 μM SFN, respectively), MDA MB 468 (90, 30 and 20 % of DMSO-treated control cells in cells treated with 10, 20 and 40 μM SFN, respectively) and SKBR-3 cells (60, 50 and 10 % of DMSO-treated cells in cells treated with 10, 20 and 40 μM SFN, respectively). In MCF-7 cells, SFN at all used concentrations inhibited Akt phosphorylation only by about 10–20 % compared with untreated controls (Fig. 4).

### Sulforaphane inhibits protein synthesis in breast cancer cells

**Fig. 5:**
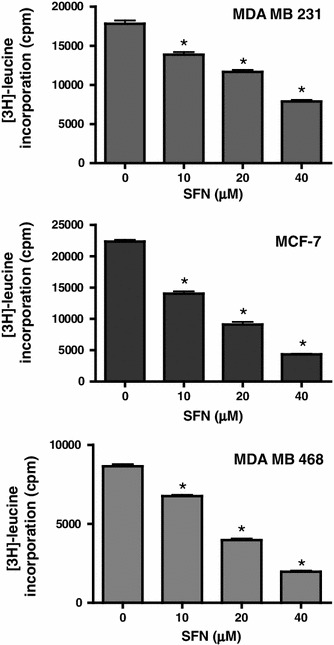
SFN inhibits protein synthesis in human breast cancer cells. MDA MB 231, MCF-7, MDA MB 468 were treated with various concentrations of SFN for 3 h in the presence of a protein precursor, [^3^H] –leucine. Cells were harvested, and radioactivity of TCA-precipitable material was estimated as described in “Materials and methods”. Results shown are mean ± SE of two independent experiments performed in duplicate (MDA MB 231 and MCF-7) or in triplicate (MDA MB 468), *significantly different (*P* < 0.01) compared with DMSO-treated control by one-way ANOVA followed by Dunnett’s multiple comparison test

Recently, we reported that SFN inhibited mTOR-S6K1 signaling and translation process in PC-3 prostate and SKBR-3 breast cancer cell lines [30]. Results presented in previous subsections of this work suggest that SFN inhibits mTOR signaling in other breast cancer cells. As mTOR is a master activator of mRNA translation, we investigated the impact of SFN on protein synthesis in MDA MB 231, MCF-7 and MDA MB 468 cells. We measured [^3^H]-leucine incorporation in control, DMSO-treated cells and cells exposed to 10, 20 or 40 μM SFN for 3 h. As demonstrated in Fig. 5, SFN inhibited [^3^H]-leucine incorporation in a dose-dependent manner. Protein synthesis in MDA MB 231 cells measured after 3-h exposure to 10, 20 or 40 μM SFN dropped to about 75, 67 and 44 % of the level seen in control cells, respectively. Similarly, in MCF-7 translation decreased to about 60, 40 and 20 % and in MDA MB 468 to 78, 40 and 20 % of control cells level upon treatment with 10, 20 or 40 μM SFN, respectively (Fig. 5).

## Discussion

In this work, we found that sulforaphane efficiently decreases survival of phenotypically distinct breast cancer cell lines which differ in the expression pattern of growth factor or estrogen receptors and PTEN suppressor (Table 1). Although anti-proliferative activity of SFN against different breast cancer cell lines has already been reported, this is the first study exploring the effect of SFN on AktmTOR-S6K1 pro-survival pathway.

Previously, it has been reported that SFN at 15 μM concentration caused accumulation of MCF-7 and MDA MB 231 cells in G2/M phase of the cell cycle after 24 h treatment and longer incubation time resulted in apoptosis initiated by mitochondrial or death receptor pathway, respectively. Moreover, global HDAC activity, as determined with an in vitro activity assay, was inhibited by SFN in MCF-7, MDA MB 231 and, to a lesser extend, in MDA MB 468 and T47D cell lines [16]. Authors also reported that IC50 of SFN did not differ significantly between these cell lines when treated for 48 h [16]. Increased tubulin acetylation and suppression of microtubules dynamic instability was observed in MCF-7 exposed to 15, 25 or 50 μM SFN [17]. G2/M cell cycle arrest of MDA MB 231 cells was associated with increased p21 and p27 cdk-cyclin inhibitors and decreased levels of cyclin A, B1 and cdc2, and apoptosis was accompanied by decreased Bcl-2 and increased caspase-3 level [31]. The same authors showed that SFN induced autophagy in MDA MB 231 cells and this process played a protective role as its inhibition by bafilomycin A1 significantly enhanced SFN-induced apoptosis [31]. It was also reported that sensitivity of breast cancer cells to SFN is connected with upregulation of p38 MAP kinase and caspase -7 activation in MCF-7 cells [32], global changes in gene expression [33] or downregulation of ER, EGFR or HER2 mRNAs [16, 34]. Other studies indicate that SFN-induced apoptosis of MCF-7 and MDA MB 231 cells is initiated by reactive oxygen species due to p66Shc translocation to mitochondria and collapse of mitochondrial membrane potential [35]. Interestingly, non-tumorigenic human mammary epithelial MCF-10A cells were resistant to SFN-induced oxidative stress and cell death [35]. Thus, it seems that cytotoxic effect of SFN is specific for cancer cells. Other reports confirm it. For instance, human mammary epithelial cell line, MCF-12A, was significantly more resistant to 48-h treatment with SFN comparing to cancer cells, MCF-7 wt and MCF-7/Adr (IC50 = 40.5 μM for MCF-12A as compared to 27.9 μM for MCF-7 wt and 13.7 μM for MCF-7/Adr) [36]. Moreover, 30 μM SFN inhibited MCF-7 and ZR75-1 cancer cells proliferation by 80 % after 48-h exposure, while proliferation of non-tumorigenic mammary cells, MCF-10F, was inhibited by about 50 % as compared to the respective controls [34]. Dose- and time-dependent growth inhibition with SFN was observed in MCF-7 and MDA 231 cells, while MCF-10A epithelial cells were more resistant, even to higher (above 10 μM) SFN concentrations. Interestingly, authors found that SFN-induced apoptosis in breast cancer cells was mediated by epigenetic regulation of telomerase gene expression [37]. None of the above mentioned research explored connection of SFN activity with AktmTOR-S6K1 pathway in breast cancer cells.

Signaling at the AktmTOR-S6K1 level is aberrantly activated in most human cancers. It is not surprising taking into account that all these kinases stimulate pro-survival processes. mTOR (the mammalian target of rapamycin) is a crucial regulator of translation and together with associated proteins, raptor, GβL, PRAS40 and DEPTOR, phosphorylates the eukaryotic initiation factor 4E-binding protein 1 (4E-BP1), an inhibitor of cap-dependent translation, and the S6 kinase 1 which positively regulates different stages of protein synthesis [38]. In addition to the role in translation, mTOR regulates the mRNA stability of some crucial cell cycle regulators, such as D cyclins or p27 cyclin-dependent kinase inhibitor as well as increases production of HIF1α, a transcription factor promoting expression of glycolytic genes [39].

S6K1 plays a pleiotropic role in translation. It participates in ribosomal biogenesis, initiation of mRNA translation through phosphorylation of eukaryotic translation initiation factor 4B (eIF4B) and regulates translation elongation through phosphorylation of eukaryotic elongation factor 2 kinase (eEF2K) [40–42]. Besides, S6K1 plays crucial role in cell growth, progression of cell cycle and cell survival inactivating pro-apoptotic proteins and stimulating synthesis of anti-apoptotic survivin [43]. S6K1 gene is amplified in 9 % of primary breast cancers which is associated with aggressive disease and poor prognosis of patients [44]. Moreover, S6K1 phosphorylates ERα leading to its transcriptional activation, which may contribute to breast cancer progression [45].

Both mTOR and S6K1 activities may be stimulated by Akt kinase. It inactivates the tuberous sclerosis TSC1/2 inhibitors of mTOR. Importantly, Akt activates other pro-survival pathways. For instance, it inhibits pro-apoptotic proteins such as Bad or transcription factors of FOXO family, promotes p53 degradation and indirectly activates pro-survival NF-κβ transcription factor [46]. AktmTOR-S6K1 are located downstream of PI3K, which is activated by membrane receptors, including the family of EGF receptors and the estrogen receptor. Hyperactivation of Akt due to activating mutations, gene amplification or enhanced signaling from receptors seems to be genetically selected during tumorigenesis and was found in many human cancers including carcinomas, glioblastoma multiforme and various hematological malignancies (reviewed in [47]). Akt not only stimulates cancer cell growth and viability but also leads to resistance to chemotherapeutics, particularly these targeting only one element of the pathway. For instance, drugs targeting mTOR-S6K1 at the same time may stimulate Akt due to inhibition of negative feedback loop where active S6K1 blocks growth factor-PI3K-Akt signaling [48]. Moreover, cancer cells treated with inhibitors of growth factor or estrogen receptors often become resistant to therapy which is caused by changes in downstream signaling components, such as activating mutations of PI3K or Akt genes and loss of suppressors, such as PTEN. Thus, agents targeting this pathway at multiple levels might be a promising alternative. Indeed, preclinical studies have shown that the mTOR antagonists can restore endocrine sensitivity in breast cancer cells [6].

Here, we show that SFN, isothiocyanate present in edible plants, targets the pro-survival pathway in breast cancer cells in at least two levels: Akt activation, determined as a drop in phosphorylation in the position crucial for the kinase activity, and mTOR-S6K1 signaling, determined as a decrease in S6K1 phosphorylation in the position recognized by mTOR (Fig. 4). Inhibition of protein synthesis (Fig. 5 and [30]) and induction of autophagy (Figs. 2, 3), both controlled by mTOR, confirm that SFN inhibits this pathway. We did not explore here the role of autophagy in the response of breast cancer cells to SFN. Even if it plays protective role such as in MDA MB 231 [31] or prostate PC-3 cells [27], it delays but not suppresses cell death.

The argument for parallel, rather than linear inhibition of Akt and mTOR by SFN, is different dephosphorylation pattern of Akt and S6K1 kinases upon SFN treatment shown on immunoblotting (Fig. 4). Moreover, Akt activation is initiated by translocation to the plasma membrane mediated by docking of the PH domain in the N-terminal region of AKT to PI(3,4,5) P_3_ on the membrane, which results in a conformational change in Akt, exposing two critical amino acid residues (serine 473 and threonine 308) for phosphorylation [49]. On the other hand, generation of PI(3,4,5) P_3_ by PI3K is inhibited by mTOR-S6K1 signaling [48]. Thus, drop in p-S6K1 should rather stimulate Akt activation if SFN targets only one signal transducer. Interestingly, previous studies on sensitivity of the panel of breast cancer cell lines to mTOR inhibitor, CCI-779, revealed that cells sensitive to CCI-779 were estrogen receptor positive, overexpressed ErbB2 and/or had lost the tumor suppressor gene product PTEN. On the other hand, resistant cell lines (such as MDA MB 231) shared none of these properties [50]. In our model, SFN inhibits viability of all tested cell lines, including MDA MB 231, which also might indicate that it acts in at least two levels.

Taken together, our data indicate that SFN might be a good therapeutics for breast cancers with different alterations of the PI3K-Ak-tmTOR-S6K1 pathway as it targets downstream elements of this pathway.

## References

[1] Pegram MD, Pauletti G, Slamon DJ (1998). HER-2/neu as a predictive marker of response to breast cancer therapy. Breast Cancer Res Treat.

[2] Slamon DJ, Clark GM, Wong SG, Levin WJ, Ullrich A, McGuire WL (1987). Human breast cancer: correlation of relapse and survival with amplification of the HER-2/neu oncogene. Science.

[3] Bianco R, Shin I, Ritter CA, Yakes FM, Basso A, Rosen N, Tsurutani J, Dennis PA, Mills GB, Arteaga CL (2003). Loss of PTEN/MMAC1/TEP in EGF receptor-expressing tumor cells counteracts the antitumor action of EGFR tyrosine kinase inhibitors. Oncogene.

[4] Bose S, Crane A, Hibshoosh H, Mansukhani M, Sandweis L, Parsons R (2002). Reduced expression of PTEN correlates with breast cancer progression. Hum Pathol.

[5] Simoncini T, Hafezi-Moghadam A, Brazil DP, Ley K, Chin WW, Liao JK (2000). Interaction of oestrogen receptor with the regulatory subunit of phosphatidylinositol-3-OH kinase. Nature.

[6] deGraffenried LA, Friedrichs WE, Russell DH, Donzis EJ, Middleton AK, Silva JM, Roth RA, Hidalgo M (2004). Inhibition of mTOR activity restores tamoxifen response in breast cancer cells with aberrant Akt activity. Clin Cancer Res.

[7] Fahey JW, Zhang Y, Talalay P (1997). Broccoli sprouts: an exceptionally rich source of inducers of enzymes that protect against chemical carcinogens. Proc Natl Acad Sci USA.

[8] Zhang Y, Kensler TW, Cho CG, Posner GH, Talalay P (1994). Anticarcinogenic activities of sulforaphane and structurally related synthetic norbornyl isothiocyanates. Proc Natl Acad Sci U S A.

[9] Singletary K, MacDonald C (2000). Inhibition of benzo[a]pyrene- and 1,6-dinitropyrene-DNA adduct formation in human mammary epithelial cells bydibenzoylmethane and sulforaphane. Cancer Lett.

[10] Zhang Y, Talalay P, Cho CG, Posner GH (1992). A major inducer of anticarcinogenic protective enzymes from broccoli: isolation and elucidation of structure. Proc Natl Acad Sci U S A.

[11] Barcelo S, Gardiner JM, Gescher A, Chipman JK (1996). CYP2E1-mediated mechanism of anti-genotoxicity of the broccoli constituent sulforaphane. Carcinogenesis.

[12] Gamet-Payrastre L, Li P, Lumeau S, Cassar G, Dupont MA, Chevolleau S, Gasc N, Tulliez J, Terce F (2000). Sulforaphane, a naturally occurring isothiocyanate, induces cell cycle arrest and apoptosis in HT29 human colon cancer cells. Cancer Res.

[13] Singh SV, Herman-Antosiewicz A, Singh AV, Lew KL, Srivastava SK, Kamath R, Brown KD, Zhang L, Baskaran R (2004). Sulforaphane-induced G2/M phase cell cycle arrest involves checkpoint kinase 2-mediated phosphorylation of cell division cycle 25C. J Biol Chem.

[14] Singh SV, Srivastava SK, Choi S, Lew KL, Antosiewicz J, Xiao D, Zeng Y, Watkins SC, Johnson CS, Trump DL, Lee YJ, Xiao H, Herman-Antosiewicz A (2005). Sulforaphane-induced cell death in human prostate cancer cells is initiated by reactive oxygen species. J Biol Chem.

[15] Jackson SJ, Singletary KW (2004). Sulforaphane inhibits human MCF-7 mammary cancer cell mitotic progression and tubulin polymerization. J Nutr.

[16] Pledgie-Tracy A, Sobolewski MD, Davidson NE (2007). Sulforaphane induces cell type-specific apoptosis in human breast cancer cell lines. Mol Cancer Ther.

[17] Azarenko O, Okouneva T, Singletary KW, Jordan MA, Wilson L (2008). Suppression of microtubule dynamic instability and turnover in MCF7 breast cancer cells by sulforaphane. Carcinogenesis.

[18] Xiao D, Powolny AA, Antosiewicz J, Hahm ER, Bommareddy A, Zeng Y, Desai D, Amin S, Herman-Antosiewicz A, Singh SV (2009). Cellular responses to cancer chemopreventive agent D, L-sulforaphane in human prostate cancer cells are initiated by mitochondrial reactive oxygen species. Pharm Res.

[19] Choi WY, Choi BT, Lee WH, Choi YH (2008). Sulforaphane generates reactive oxygen species leading to mitochondrial perturbation for apoptosis in human leukemia U937 cells. Biomed Pharmacother.

[20] Myzak MC, Hardin K, Wang R, Dashwood RH, Ho E (2006). Sulforaphane inhibits histone deacetylase activity in BPH-1, LnCaP and PC-3 prostate epithelial cells. Carcinogenesis.

[21] Mi L, Gan N, Cheema A, Dakshanamurthy S, Wang X, Yang DC, Chung FL (2009). Cancer preventive isothiocyanates induce selective degradation of cellular alpha- and beta-tubulins by proteasomes. J Biol Chem.

[22] Jackson SJ, Singletary KW (2004). Sulforaphane: a naturally occurring mammary carcinoma mitotic inhibitor, which disrupts tubulin polymerization. Carcinogenesis.

[23] Konecny GE, Pegram MD, Venkatesan N, Finn R, Yang G, Rahmeh M, Untch M, Rusnak DW, Spehar G, Mullin RJ, Keith BR, Gilmer TM, Berger M, Podratz KC, Slamon DJ (2006). Activity of the dual kinase inhibitor lapatinib (GW572016) against HER-2-overexpressing and trastuzumab-treated breast cancer cells. Cancer Res.

[24] Lu Y, Lin YZ, LaPushin R, Cuevas B, Fang X, Yu SX, Davies MA, Khan H, Furui T, Mao M, Zinner R, Hung MC, Steck P, Siminovitch K, Mills GB (1999). The PTEN/MMAC1/TEP tumor suppressor gene decreases cell growth and induces apoptosis and anoikis in breast cancer cells. Oncogene.

[25] Yarden RI, Lauber AH, El-Ashry D, Chrysogelos SA (1996). Bimodal regulation of epidermal growth factor receptor by estrogen in breast cancer cells. Endocrinology.

[26] Watkins SC, Cullen MJ (1987). A qualitative and quantitative study of the ultrastructure of regenerating muscle fibres in Duchenne muscular dystrophy and polymyositis. J Neurol Sci.

[27] Herman-Antosiewicz A, Johnson DE, Singh SV (2006). Sulforaphane causes autophagy to inhibit release of cytochrome C and apoptosis in human prostate cancer cells. Cancer Res.

[28] Levine B, Klionsky DJ (2004). Development by self-digestion: molecular mechanisms and biological functions of autophagy. Dev Cell.

[29] Manning BD, Tee AR, Logsdon MN, Blenis J, Cantley LC (2002). Identification of the tuberous sclerosis complex-2 tumor suppressor gene product tuberin as a target of the phosphoinositide 3-kinase/akt pathway. Mol Cell.

[30] Wiczk A, Hofman D, Konopa G, Herman-Antosiewicz A (2012). Sulforaphane, a cruciferous vegetable-derived isothiocyanate, inhibits protein synthesis in human prostate cancer cells. Biochim Biophys Acta.

[31] Kanematsu S, Uehara N, Miki H, Yoshizawa K, Kawanaka A, Yuri T, Tsubura A (2010). Autophagy inhibition enhances sulforaphane-induced apoptosis in human breast cancer cells. Anticancer Res.

[32] Jo EH, Kim SH, Ahn NS, Park JS, Hwang JW, Lee YS, Kang KS (2007). Efficacy of sulforaphane is mediated by p38 MAP kinase and caspase-7 activations in ER-positive and COX-2-expressed human breast cancer cells. Eur J Cancer Prev.

[33] Telang U, Brazeau DA, Morris ME (2009). Comparison of the effects of phenethyl isothiocyanate and sulforaphane on gene expression in breast cancer and normal mammary epithelial cells. Exp Biol Med (Maywood).

[34] Ramirez MC, Singletary K (2009). Regulation of estrogen receptor alpha expression in human breast cancer cells by sulforaphane. J Nutr Biochem.

[35] Sakao K, Singh SV (2012). D, L-sulforaphane-induced apoptosis in human breast cancer cells is regulated by the adapter protein p66Shc. J Cell Biochem.

[36] Tseng E, Scott-Ramsay EA, Morris ME (2004). Dietary organic isothiocyanates are cytotoxic in human breast cancer MCF-7 and mammary epithelial MCF-12A cell lines. Exp Biol Med (Maywood).

[37] Meeran SM, Patel SN, Tollefsbol TO (2010). Sulforaphane causes epigenetic repression of hTERT expression in human breast cancer cell lines. PLoS ONE.

[38] Mamane Y, Petroulakis E, LeBacquer O, Sonenberg N (2006). mTOR, translation initiation and cancer. Oncogene.

[39] Magnuson B, Ekim B, Fingar DC (2012). Regulation and function of ribosomal protein S6 kinase (S6K) within mTOR signalling networks. Biochem J.

[40] Holz MK, Ballif BA, Gygi SP, Blenis J (2005). mTOR and S6K1 mediate assembly of the translation preinitiation complex through dynamic protein interchange and ordered phosphorylation events. Cell.

[41] Wang X, Li W, Williams M, Terada N, Alessi DR, Proud CG (2001). Regulation of elongation factor 2 kinase by p90(RSK1) and p70 S6 kinase. EMBO J.

[42] Raught B, Peiretti F, Gingras AC, Livingstone M, Shahbazian D, Mayeur GL, Polakiewicz RD, Sonenberg N, Hershey JW (2004). Phosphorylation of eucaryotic translation initiation factor 4B Ser422 is modulated by S6 kinases. EMBO J.

[43] Vaira V, Lee CW, Goel HL, Bosari S, Languino LR, Altieri DC (2007). Regulation of survivin expression by IGF-1/mTOR signaling. Oncogene.

[44] Wu GJ, Sinclair CS, Paape J, Ingle JN, Roche PC, James CD, Couch FJ (2000). 17q23 amplifications in breast cancer involve the PAT1, RAD51C, PS6K, and SIGma1B genes. Cancer Res.

[45] Yamnik RL, Digilova A, Davis DC, Brodt ZN, Murphy CJ, Holz MK (2009). S6 kinase 1 regulates estrogen receptor alpha in control of breast cancer cell proliferation. J Biol Chem.

[46] Manning BD, Cantley LC (2007). AKT/PKB signaling: navigating downstream. Cell.

[47] Altomare DA, Testa JR (2005). Perturbations of the AKT signaling pathway in human cancer. Oncogene.

[48] Shah OJ, Wang Z, Hunter T (2004). Inappropriate activation of the TSC/Rheb/mTOR/S6K cassette induces IRS1/2 depletion, insulin resistance, and cell survival deficiencies. Curr Biol.

[49] Stephens L, Anderson K, Stokoe D, Erdjument-Bromage H, Painter GF, Holmes AB, Gaffney PR, Reese CB, McCormick F, Tempst P, Coadwell J, Hawkins PT (1998). Protein kinase B kinases that mediate phosphatidylinositol 3,4,5-trisphosphate-dependent activation of protein kinase B. Science.

[50] Yu K, Toral-Barza L, Discafani C, Zhang WG, Skotnicki J, Frost P, Gibbons JJ (2001). mTOR, a novel target in breast cancer: the effect of CCI-779, an mTOR inhibitor, in preclinical models of breast cancer. Endocr Relat Cancer.

